# Facial Dysmorphism and Severe Vascular Phenotype in TRNT1 Deficiency with Concomitant Antithrombin III Deficiency

**DOI:** 10.1007/s10875-026-02047-5

**Published:** 2026-06-27

**Authors:** David M. Matea, Smaranda T. Arghirescu, Adela Chirita-Emandi, Patricia Urtila, Mihaela Bataneant

**Affiliations:** 1 Doctoral School, ‛Victor Babeș’ University of Medicine and Pharmacy of Timisoara, Timisoara, 300041 Romania; 2 Clinical Emergency Hospital for Children “Louis Țurcanu”, Timișoara, Romania; 3 Department of Pediatrics, University of Medicine and Pharmacy “Victor Babeș”, Timișoara, Romania; 4 Department of Microscopic Morphology-Genetics, Centre for Genomic Medicine, University of Medicine and Pharmacy “Victor Babeș”, Timișoara, Romania; 5 Regional Center of Medical Genetics Timiș, Timișoara, Romania

## Abstract

TRNT1 deficiency (SIFD syndrome) is a rare inborn error of immunity characterized by sideroblastic anemia, immunodeficiency, periodic fevers, and developmental delay. We report two Romanian patients with genetically confirmed TRNT1 deficiency presenting with characteristic hematologic and immunologic abnormalities and a distinctive facial dysmorphism. One patient additionally developed severe gastrointestinal ischemia and intracranial hemorrhage in the setting of concomitant hereditary antithrombin III deficiency. These observations expand the phenotypic spectrum associated with TRNT1 deficiency and highlight the marked clinical variability of this disorder. The contribution of TRNT1-associated inflammation to the vascular phenotype remains speculative and cannot be distinguished from the effects of the concomitant thrombophilic condition.

To the Editor,

TRNT1 deficiency (SIFD syndrome; OMIM #616084) is a rare autosomal recessive inborn error of immunity characterized by sideroblastic anemia, B-cell immunodeficiency, periodic fevers, and developmental delay, with marked phenotypic heterogeneity [1, 2]. We report two Romanian patients with genetically confirmed TRNT1 deficiency presenting with characteristic hematologic and immunologic abnormalities, a distinctive facial dysmorphism, and, in one patient, severe vascular manifestations in the setting of concomitant hereditary antithrombin III (ATIII) deficiency [3].

Patient 1 presented during infancy with recurrent infections, developmental delay, transfusion-dependent microcytic sideroblastic anemia, and persistent inflammatory manifestations. Physical examination revealed facial dysmorphism characterized by frontal bossing, midface hypoplasia, and downslanting palpebral fissures. Laboratory evaluation confirmed sideroblastic anemia and immune dysregulation with hypogammaglobulinemia. Genetic testing, including whole-exome sequencing, identified compound heterozygous TRNT1 variants c.608 + 1G > T and c.1246 A > G (p.Lys416Glu). Additional features included congenital bilateral cataract, strabismus, congenital cardiac defects, and global developmental delay. Despite supportive treatment, the patient developed progressive multisystem disease and died following COVID-19 infection.

Patient 2 presented with recurrent infections, severe anemia, growth failure, and inflammatory episodes beginning early in life. Facial dysmorphism similar to that of Patient 1 was documented. Genetic testing identified compound heterozygous TRNT1 variants c.428_431del (p.Asp143Glyfs*12) and c.1246 A > G (p.Lys416Glu); concurrent testing identified hereditary ATIII deficiency. During follow-up, the patient developed severe gastrointestinal ischemia with portal venous gas and pneumatosis intestinalis on abdominal computed tomography, requiring extensive intestinal resection. The clinical course was further complicated by intracranial hemorrhage with extensive subdural hematoma and subarachnoid hemorrhage (Fig. 1).

**Fig. 1:**
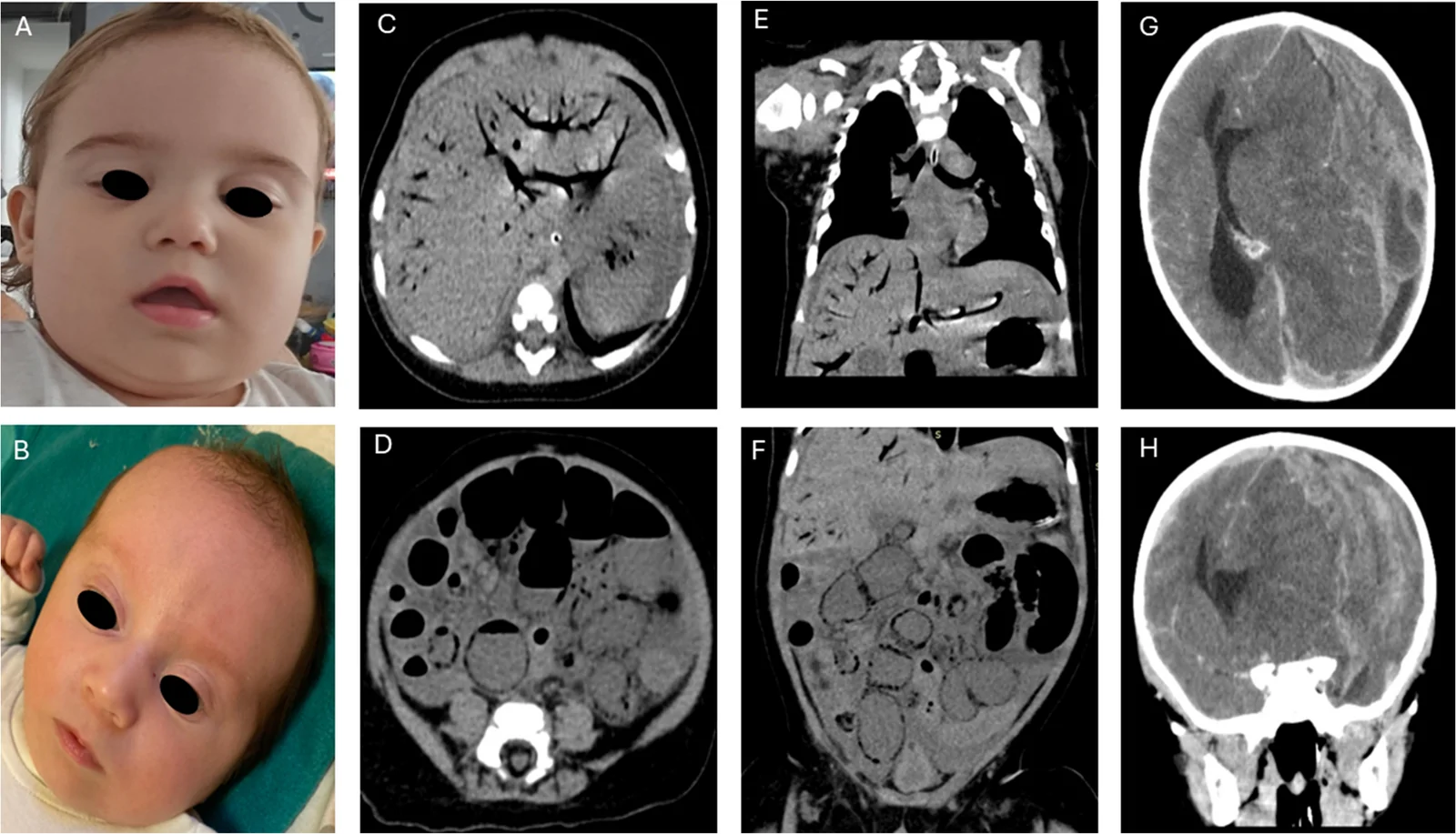
Clinical and radiologic features in patients with TRNT1 deficiency. (**A**–**B**) Facial dysmorphism observed in the two patients, demonstrating frontal bossing, midface hypoplasia, and downslanting palpebral fissures. (**C**–**F**) Abdominal CT imaging demonstrating portal venous gas, pneumatosis intestinalis, and dilated bowel loops consistent with severe bowel ischemia. (**G**–**H**) Cranial CT demonstrating extensive subdural hematoma with significant mass effect and associated subarachnoid hemorrhage

The coexistence of TRNT1 deficiency and hereditary ATIII deficiency in Patient 2 complicates the attribution of the vascular phenotype. While ATIII deficiency likely represented the principal thrombotic risk factor, the unusually aggressive vascular course raises the possibility that TRNT1-associated systemic inflammation [4] may have contributed to disease severity. However, causality cannot be established from isolated clinical observations. Notably, Patient 2 maintained a normal absolute B-cell count despite a severe and ultimately fatal disease course, consistent with emerging data suggesting that preserved B-cell counts do not preclude severe outcomes [5].

Our observations expand the phenotypic spectrum of TRNT1 deficiency by describing a distinctive facial dysmorphism in two unrelated patients with normal karyotype and chromosomal microarray analyses, and by reporting, to our knowledge, the first described association with hereditary ATIII deficiency. These findings underscore the marked clinical variability of TRNT1 deficiency and support careful clinical evaluation of severe vascular manifestations occurring in the context of systemic inflammation and concomitant thrombophilic conditions.

This report is limited by the small number of patients, the lack of functional studies of inflammatory and coagulation pathways, and the inability to definitively attribute the vascular phenotype to TRNT1 deficiency in the presence of concomitant hereditary thrombophilia.

## Data Availability

The data supporting the findings of this study are included within the article. Additional information may be available from the corresponding author upon reasonable request, subject to ethical and privacy restrictions.
